# Swin-Transformer-Based YOLOv5 for Small-Object Detection in Remote Sensing Images

**DOI:** 10.3390/s23073634

**Published:** 2023-03-31

**Authors:** Xuan Cao, Yanwei Zhang, Song Lang, Yan Gong

**Affiliations:** 1 Suzhou Institute of Biomedical Engineering and Technology, Chinese Academy of Sciences, Suzhou 215613, China; 2 School of Physical Science and Technology, Suzhou University of Science and Technology, Suzhou 215009, China; 3 Jinan Guoke Medical Technology Development Co., Ltd., Jinan 250104, China

**Keywords:** Swin Transformer, YOLOv5, multi-scale feature fusion, attention mechanism, small-object detection, remote sensing

## Abstract

This study aimed to address the problems of low detection accuracy and inaccurate positioning of small-object detection in remote sensing images. An improved architecture based on the Swin Transformer and YOLOv5 is proposed. First, Complete-IOU (*CIOU*) was introduced to improve the K-means clustering algorithm, and then an anchor of appropriate size for the dataset was generated. Second, a modified CSPDarknet53 structure combined with Swin Transformer was proposed to retain sufficient global context information and extract more differentiated features through multi-head self-attention. Regarding the path-aggregation neck, a simple and efficient weighted bidirectional feature pyramid network was proposed for effective cross-scale feature fusion. In addition, extra prediction head and new feature fusion layers were added for small objects. Finally, Coordinate Attention (CA) was introduced to the YOLOv5 network to improve the accuracy of small-object features in remote sensing images. Moreover, the effectiveness of the proposed method was demonstrated by several kinds of experiments on the DOTA (Dataset for Object detection in Aerial images). The mean average precision on the DOTA dataset reached 74.7%. Compared with YOLOv5, the proposed method improved the mean average precision (*mAP*) by 8.9%, which can achieve a higher accuracy of small-object detection in remote sensing images.

## 1. Introduction

Object detection technology for remote sensing images is an important content area in computer vision and a basic task in many practical applications, such as plant protection, wildlife protection, urban traffic management, etc. [1]. However, because of the influence of intricate factors, such as dramatic scale variance and complicated backgrounds filled with distractors, small objects (usually defined as objects with pixels below 32 × 32) in remote sensing images are always missed. Therefore, the detection of small objects in remote sensing images is still a challenging task.

In recent years, convolutional neural networks (CNNs) have achieved significant progress in object detection tasks owing to their superior feature representation capabilities and the research in remote sensing images is currently dominated by them [2]. Current CNN-based object detection algorithms mainly include Faster R-CNN [3], YOLO series algorithms, SSD [4], and so on. These methods have achieved good results in natural image datasets such as MS COCO, PASCAL VOC, etc. [5]. However, when these methods are used in remote sensing images, the results are always hardly satisfactory. The main reason is that remote sensing images are obtained by sensors on aerospace and aviation equipment taken from a bird’s-eye view [6]. As a result, most remote sensing images have a wide imaging range, complex background, and an imbalanced distribution of front items [7]. Additionally, the objects in remote sensing images are always small. For instance, the mean size of objects in AI-TOD is about 12.8 pixels, which is much smaller than most of the natural datasets. Compared to large/medium-sized objects, small-sized objects often lack sufficient information about their appearance to distinguish them from complex backgrounds. Finally, the modeling locality of CNNs limits their ability to capture global contextual information in remote sensing images [8]. Generally speaking, it is hard to apply CNN-based object detection algorithms directly to remote sensing images for small-object detection.

Consequently, we modified YOLOv5 based on the Swin Transformer and propose a one-stage detection architecture for small-object detection in remote sensing images. First, Complete-IOU (*CIOU*) is introduced into K-means clustering algorithm as similar index to generate more suitable anchor boxes for remote sensing dataset. Then, the deep feature maps fused in the neck of YOLOv5 uses a simple channel-wise addition or superposition of feature maps. To avoid this issue, we supplement a small-object prediction head and introduce BiFPN to enhance the feature extraction ability of the network by learning the weights of different input features. Second, since YOLOv5 is a CNN-based detector, the modeling locality of CNN limits the ability to capture global contextual information. We proposed an improved CSPDarknet53 network based on the Swin Transformer and used in the Backbone and Neck to fully utilize CNN’s powerful feature extraction capability and the Transformer’s time series processing capability. Finally, the Coordinate Attention Module (CA) is used to improve attention performance by embedding positional information into channel attention. The experimental results on the DOTA dataset demonstrate that the detector outperforms previous detectors in detecting small objects.

The main contributions of this study are as follows.

- The improved K-means clustering algorithm makes the algorithm more suitable for the DOTA dataset, improving the recall rate and accelerating the convergence speed of the model.
- An improved CSPDarknet53 network based on the Swin Transformer (C3SW-T) is proposed, which preserves the context information better and improves the accuracy of small-target detection.
- A deeper and highly effective weighted bidirectional feature pyramid network is presented to improve the detection performance of objects of different scales, especially for small objects.
- Coordinate Attention (CA) was introduced into YOLOv5 to allow the network to pay attention to a larger area without unnecessary computational overhead.

## 2. Materials and Methods

### 2.1. Object Detection Based on CNN

At present, CNN-based object detection algorithms are mainly divided into two categories [9]: two-stage object detection methods and single-stage object detection methods.

The two-stage methods are based on region recommendation, such as Region-CNN (R-CNN) [10], Faster R-CNN [3], etc. These methods first use CNN to extract features from the candidate regions and finally apply the classifier for classification at the second stage. Due to extensive development, the detection accuracy and speed of two-stage algorithms have been greatly improved. However, two-stage methods still have large number parameters and it will make the detection process very time consuming [11].

As the two-stage object detection algorithms cannot meet the requirement of real-time detection, faster and less computationally expensive single-stage object detection algorithms were proposed, such as the YOLO series algorithms, SSD, RetinaNet, etc. These methods simplify the object detection problem into a regression problem and directly predict the class probability and regression location information based on the bounding box corresponding to each grid [12]. YOLOv5 is a superior architecture in the YOLO series algorithms, which inherits all the advantages of the previous versions and is superior in terms of detection speed and accuracy. YOLOv5 combines the structures of the improved CSPDarknet53 network, SPPF, the Path Aggregation Network (PANet), the residual network, etc. The largest model of YOLOv5 reports the highest *mAP* of 68.9% on the MS COCO dataset and is a state-of-the-art object detector. According to the network width and depth, YOLOv5 has five derived models: YOLOv5n, YOLOv5s, YOLOv5m, YOLOv5l, and YOLOv5x. The latter model has a better performance but more parameters. In order to better conduct follow-up research on model deployment, we selected YOLOv5s as the baseline model.

### 2.2. Vision Transformer

Transformers have achieved remarkable results in the fields of machine translation and natural language processing (NLP). Many scholars have applied transformers in the field of computer vision, such as ViT (Vision Transformer) [13], DETR (DEtection TRansformer) [14], and Swin Transformer (Swin-T) [15]. The emergence of these algorithms has provided new solutions to problems in the visual field and achieved good results due to their powerful ability to focus on global information modeling by directly comparing features across all spatiotemporal locations [16].

In the field of object detection, some scholars have already used the Transformer model to calculate the entire image to capture the global information of the image, such as DETR [14], Swin Transformer [15], etc. The Swin Transformer (SW-T) is a successful attempt to introduce Transformer into the object detection field. The Swin Transformer constructs a hierarchical feature representation by starting from small image fragments and gradually merging neighboring patches in deeper Transformer layers. This architecture can conveniently advance the model for dense prediction tasks and achieves impressive speed and accuracy in object detection in MS COCO.

However, although the Transformer model with global computing characteristics has a strong overall performance, it loses some local information and is insensitive to small objects, making the detection effect of small objects poor [17]. To achieve better performance, we used a combination of CNN and Transformer and integrated the Swin Transformer and YOLOv5 network to improve the detection accuracy and robustness of the model.

### 2.3. Methods

**Figure 1:**
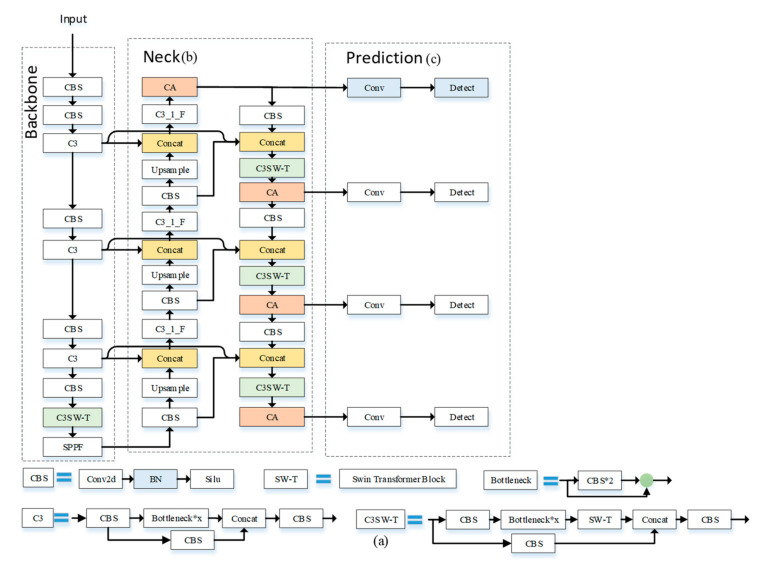
Network structure diagram of improved YOLOv5. (a) CSPDarknet53 with Swin Transformer Block at the Neck and end of Backbone. (b) The Neck uses a structure like BiFPN. (c) Four prediction heads use the feature maps from Coordinate Attention.

The framework of our proposed method for small-object detection in remote sensing images is illustrated in Figure 1. The proposed work consists of four parts: Input, Backbone, Neck, and Prediction. In Backbone, feature information from input images is extracted and three different scales of feature maps are sent to Neck to combine. In the end of Backbone, we added an improved CSPDarknet53 network based on the Swin Transformer to improve the feature extraction ability. Then, we use a bidirectional feature pyramid network and expand the feature fusion layer to a detection branch structure of four scales. In addition, various combinations of activation functions such as ReLu, Sigmod, etc. are also used in proposed work. Finally, an Coordinate Attention Mechanism is added to our network and prediction heads use the feature maps from Coordinate Attention.

#### 2.3.1. Improved K-Means Clustering Algorithm

Anchor box is an initial candidate box that has a fixed size and aspect ratio to avoid the model from blindly learning the target position and target scale during the training process [18]. For different datasets, the size and aspect ratio of the anchor box are affected by the size of all anchor boxes in the dataset [19]. Therefore, the selection of appropriate anchor boxes is necessary to stabilize model training and accelerate the convergence rate.

YOLOv5 uses standard K-means clustering instead of manually designing the anchor boxes [20]. Before the training process starts, the Euclidean Distance is used as the similarity index to cluster the bounding boxes of the training dataset and automatically generate a set of suitable anchor boxes for the dataset. The calculation formula is shown in Equation (1).

(1) $$d(box,anchor)=\rho^{2}(b^{box},b^{anchor})$$

The central points of the bounding boxes are represented by $b^{box},b^{anchor}$ and their Euclidean distance is ρ(·).

**Figure 2:**
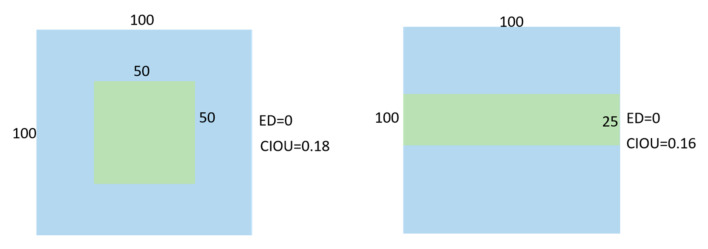
Schematic diagram of the difference between *IOU* and *CIOU*.

However, the Euclidean Distance cannot distinguish between different alignments of two bounding boxes. As shown in Figure 2, the results of Euclid Distance are both 0, but *CIOU* are 0.18 and 0.16, respectively.

To address this issue, we used *CIOU* [21] to distinguish the different alignment between two anchor boxes. The *CIOU* formulation is expressed as Equation (2):

(2) $$L_{CIOU}=1-IOU+\frac{\rho^{2}(b^{A},b^{B})}{c^{2}}+\alpha \upsilon$$

(3) $$\alpha =\frac{\upsilon }{(1-IoU)+\upsilon }$$

(4) $$\upsilon =\frac{4}{\pi^{2}}(arctan\frac{w^{A}}{h^{A}}-arctan\frac{w^{B}}{h^{B}})$$

where α is a positive trade-off parameter and υ measures the consistency of the aspect ratio. The central points of the bounding boxes are represented by $b^{A},b^{B}$ and their Euclidean distance is ρ(·).

Therefore, we used *CIOU* as the similarity index to generate the anchor boxes. The improved formula can be expressed as follows:

(5) d(box,anchor)=1−CIOU(box,anchor)

#### 2.3.2. Swin Transformer Block

To solve the problem of loss of detailed information in the process of YOLOv5 feature extraction for remote sensing images with large-scale and complex scenarios, we propose an improved CSPDarknet53 structure that integrates the Swin Transformer Block (C3SW-T). C3SW-T can expand the receptive field of the network and provides higher efficiency, better capture of global information, and enrichment of contextual information.

**Figure 3:**
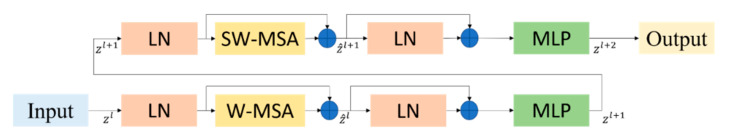
The architecture of Swin Transformer.

The structure of the Swin Transformer block is shown in Figure 3. It consists of layer normalization (LN), window-based multi-head self-attention (*W*-*MSA*), shifted window-based multi-head self-attention (*SW*-*MSA*) and a 2-layer multi-layer perceptron (*MLP*) with GELU nonlinearity in between [15]. LN helps the network fuse better and prevents the network from overfitting. *W*-*MSA* and *SW*-*MSA* can help the model to pay attention to the relevant information in other adjacent windows and perform feature interaction across windows. *MLP* can be used for feature transformation and nonlinearity composed of residual connections.

The specific process of the input feature data from level *l* to level *l* + 1 can be expressed as Equations (6)–(9).

(6) $${\hat{z}}^{l}=W-MSA(LN(z^{l-1}))+z^{l-1}$$

(7) $$z^{l}=MLP(LN({\hat{z}}^{l}))+{\hat{z}}^{l}$$

(8) $${\hat{z}}^{l+1}=SW-MSA(LN(z^{l}))+z^{l}$$

(9) $$z^{l+1}=MLP(LN({\hat{z}}^{l+1}))+{\hat{z}}^{l+1}$$

where $z^{l}$ and ${\hat{z}}^{l}$ denote the output features of the *MLP* and the two self-attention mechanisms in the first block.

**Figure 4:**
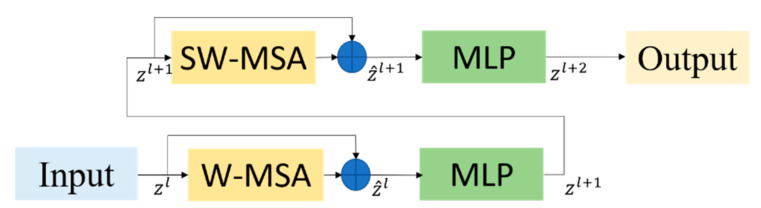
Modified Swin Transformer Block.

However, the LN layer will destroy the sample features learned when used in the CNN [22]. Therefore, we modified the Swin Transformer to make it more suitable for the YOLOv5 network. The modified Swin Transformer Block is shown in Figure 4.

The improved specific process of the input feature data from level *l* to level *l + 1* can be expressed as Equations (10)–(13).

(10) $${\hat{z}}^{l}=W-MSA(z^{l-1})+z^{l-1}$$

(11) $$z^{l}=MLP({\hat{z}}^{l})+{\hat{z}}^{l}$$

(12) $${\hat{z}}^{l+1}=SW-MSA(z^{l})+z^{l}$$

(13) $$z^{l+1}=MLP({\hat{z}}^{l+1})+{\hat{z}}^{l+1}$$

**Figure 5:**
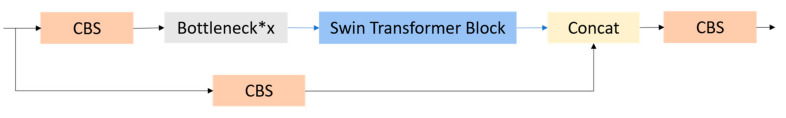
Schematic of C3SW-T.

Therefore, we introduced the Swin Transformer Block into the CSPDarknet53 network (C3SW-T) as shown in Figure 5, and replaced the C3 modules in the backbone and neck of the YOLOv5. The characteristics of the Swin Transformer were used to better capture the information that is beneficial to small objects and improve the portability of learned features simultaneously.

#### 2.3.3. Coordinate Attention

Small objects in an image occupy fewer pixels and are easily affected by complex scenarios. The original YOLOv5 algorithm easily loses the feature information of small-object when using convolutional sampling and produces limited detection results for small objects [23]. Therefore, we added the Coordinate Attention [24] structure to further improve the accuracy of small-object detection without introducing unnecessary computational overheads.

**Figure 6:**
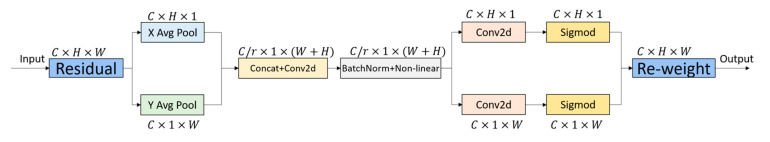
Schematic of the CA block.

The network structure is shown in Figure 6

The CA module utilizes two one-dimensional global pooling operations to aggregate vertical and horizontal input features into two independent orientation-aware feature maps. These two feature maps embedded with specific orientation information are then encoded as two attention maps. Each attention map captures an input feature with longer temporal dependencies in different spatial directions. In order to improve the expressive ability of the feature map, the location information is saved in the generated interest map, and then the two interest maps and the input feature map are multiplied.

#### 2.3.4. Multi-Scale Feature Detection

**Figure 7:**
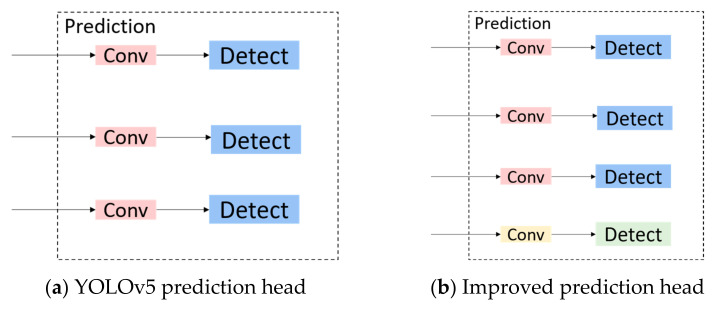
Comparison of YOLOv5 prediction layer and proposed work.

Three different sizes of feature maps were used to detect objects of different sizes in YOLOv5, as shown in Figure 7a. However, when the width or height of the object in the image is less than 8 pixels, insufficient feature learning occurs which leads to missed detection. If the number of images is enormous and only simple downsampling is performed, the downsampling multiple will be too large and data information will easily be lost. However, if the number is too small, the forward propagation of the network needs to save many feature maps in memory, which exhausts GPU resources and is prone to memory explosion, making it impossible for normal training and reasoning [25].

Therefore, we added a multi-scale feature extraction layer and a feature fusion layer after its original feature extraction layer and expanded it to a detection branch structure of four scales, as shown in Figure 7b. The network uses YOLOv5 to extract feature information in a deeper network for enhancing the multi-scale learning ability of the model in drone-captured scenarios and to better learn the multi-level feature information of the target and improve the small target detection performance in drone-captured scenarios.

#### 2.3.5. Improvement of Feature Fusion Network

YOLOv5 uses a simple channel-wise addition or superposition of feature maps of different resolutions during feature fusion. The feature fusion network accelerates the flow of underlying information [1] and can bidirectionally fuse the strong semantic information of the high-level feature map with the weak semantic information of the low-level feature map, significantly improving the accuracy of target detection [26,27]. The YOLOv5 feature fusion process is shown in Equations (14) and (15).

(14) Pitd=Conv(Piin+Resize(Pi+1td))

(15) Piout=Conv(Pitd+Resize(Pi−1out))

where $P_{i}^{in}$ represents the feature at level *i* in the backbone, $P_{i}^{out}$ represents the output feature of the neck, and $P_{i}^{td}$ represents the intermediate feature generated by the Neck layer. Conv is usually a series of convolution operations related to feature processing, and the Resize operation is used to adjust the size of the feature map to fit the size of the current feature layer.

**Figure 8:**
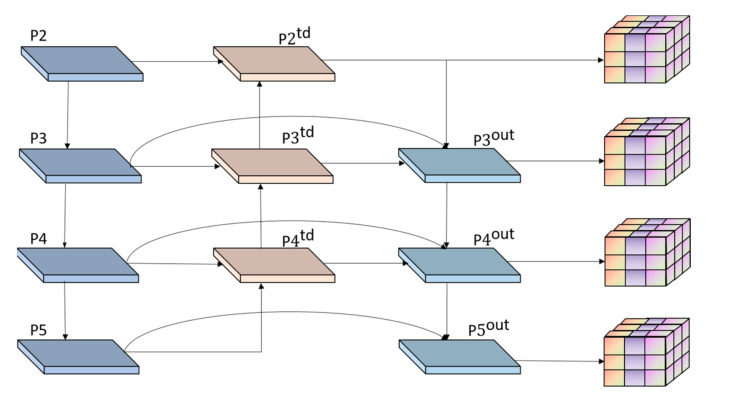
Improved feature fusion network.

However, YOLOv5 does not consider that the contribution of feature maps of different resolutions to the fusion input features as equal [28]. Therefore, we reconcile the idea of the weighted bidirectional feature pyramid and designed the bidirectional feature pyramid network, as shown in Figure 8. The improved network introduces bidirectional cross-scale connections and fast normalization fusion with learnable weights to learn the weights of different input features and simultaneously improve the feature representation capability of the network concurrently.

The improved feature fusion formula is expressed as Equation:

(16) Pitd=Conv(Piin+Resize(Pi+1td)w1+w2+ε)

(17) Piout=Conv(w1'Piin+w2'Resize(Pitd)+w3'Resize(Pi−1out)w1+w2+w3+ε)

where $w_{i}$ is a learnable weight representing the importance of the corresponding feature. The larger the value, the greater the influence of the feature on the final fusion result. ε is a small value that prevents the numerator from being zero.

## 3. Results

### 3.1. Dataset

**Figure 9:**
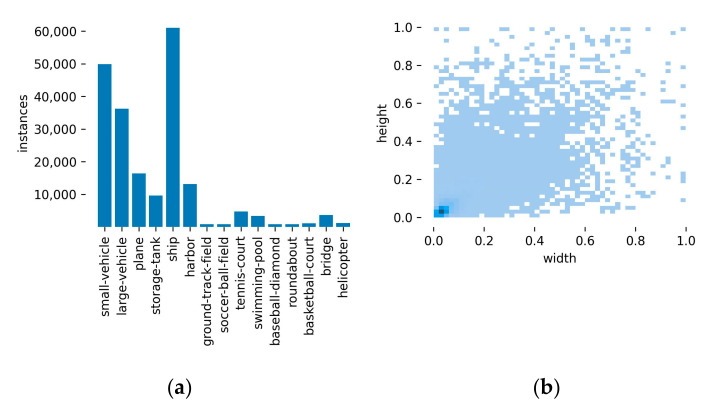
(**a**) The distribution of the classes of the labels; (**b**) heat map of the size.

DOTA [29] is a widely used high-resolution remote sensing dataset with 15 common object categories: Plane (*PL*), ground field track (GFT), small vehicles (SV), etc. There are 2806 images with a size of 4k × 4k pixels and 188,282 labels in the DOTA dataset. Figure 9 shows the distribution of classifications and the size of objects in the DOTA dataset. As shown in Figure 9, the DOTA dataset contains vast unbalanced samples and small objects that are barely over 5% of the image size. Because the image size in the DOTA dataset was too large, it could not be directly input into the network model for training. Therefore, we preprocessed the images in the DOTA dataset and divided them into sub-images with a pixel size of 1024 × 1024. In this paper, we used 15,749 images for training, 5297 images for validation, and 12,779 images for testing.

### 3.2. Experimental Environment and Parameter Settings

The experimental training environment was configured as follows: Intel Core i5-10400F, all models were trained on NVIDIA RTX 3070 and tested on NVIDIA RTX 2060S, and the program was implemented under Windows10, Pytorch framework, and CUDA Toolkit 11.2.

The training used the SGD algorithm to optimize the loss function. The momentum was set to 0.937, the weight decay coefficient to 0.0005, the initial learning rate to 0.01, the batch size to 32, the resolution of the input image to 640 × 640, and other parameter settings were consistent with the default settings of YOLOv5. In addition, we used optimization strategies, such as warm-up training, cosine annealing, gradient accumulation, and exponential moving average.

### 3.3. Evaluation Indicators

After model training was completed, the trained weights were used to test the model, and the model was evaluated from multiple aspects. The performance of the model was evaluated using precision, recall, and mean average precision (*mAP*). Precision is the ratio of predicted positive examples to all detected objects, and recall rate is the ratio of the number of correctly detected objects to the number of all labeled objects. The calculation formula is as follows:

(18) $$P=\frac{TP}{TP+FP}\times 100\%$$

(19) $$R=\frac{TP}{TP+FN}\times 100\%$$

where *TP* is the number of targets detected correctly by the model, *FP* is the number of targets detected incorrectly by the model, and *FN* is the number of correct targets missed by the model. The area of the *P*-*R* curve is the average precision *AP* (Average Precision, *AP*) of the category, and its calculation formula is as follows:

(20) $$AP=\int_{0}^{1}P(R)dR$$

(21) $$mAP=\frac{1}{c}\sum_{i=1}^{c}AP_{i}=\frac{1}{c}\int_{0}^{1}P(R)dR$$

where *c* is the number of categories in the multiclass detection task, and $AP_{i}$ is the average accuracy of each *i*-th category of objects.

### 3.4. Training Results

**Figure 10:**
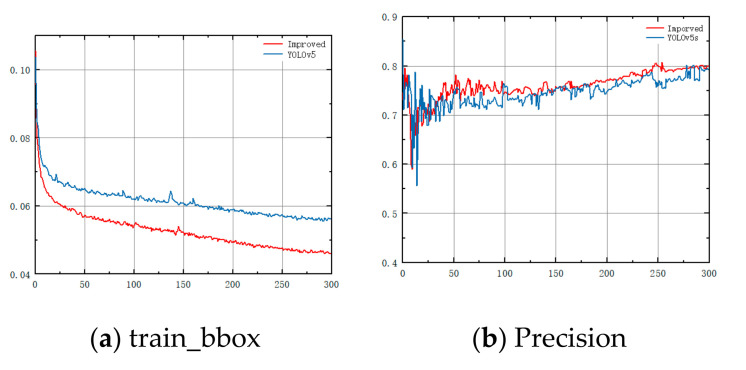

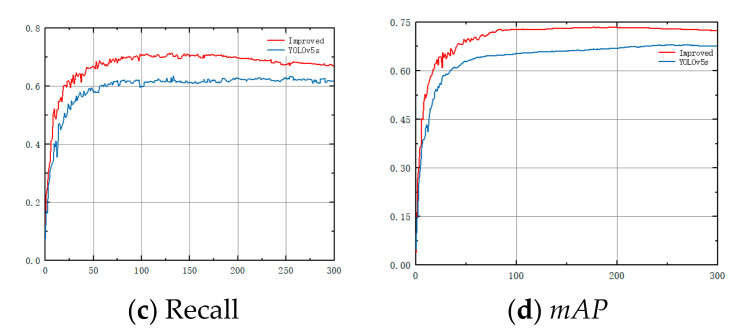
Training curve of this article and YOLOv5.

Figure 10 shows the changes in the common indicators of the improved algorithm and YOLOv5s training process. Figure 10a shows the performance curve of the confidence loss for the training. As can be seen, the confidence loss of our method converges faster and gradually decreases to 0.04, which is always lower than that of YOLOv5s. The other graphs in Figure 10 are the performance curves for precision, recall, and *mAP*, respectively. These indicators can measure the performance of the model in classification problems. The higher the value, the higher the detection accuracy of the model is. As the number of training sessions increased, the training metrics of the model improved gradually. Figure 10b–d shows that the precision, recall, and *mAP* were higher than those of YOLOv5s and reached a stable state after fewer training times. The training speed and efficiency of the proposed method were also faster than those of YOLOv5s.

## 4. Discussions

### 4.1. Compared with YOLOv5

Table 1 lists the *AP* value of each category and the *mAP* values of all the categories for YOLOv5s and the proposed work. Compared with YOLOv5, the *mAP* of the algorithm in this study increased by 6.8%, and the *AP* values of 14 categories improved, among which the *AP* values of ST, *SBF*, BD, and *BR* increased by more than 10%. This indicates that the proposed work could greatly improve the accuracy of small-object detection, which means better performance.

**Figure 11:**
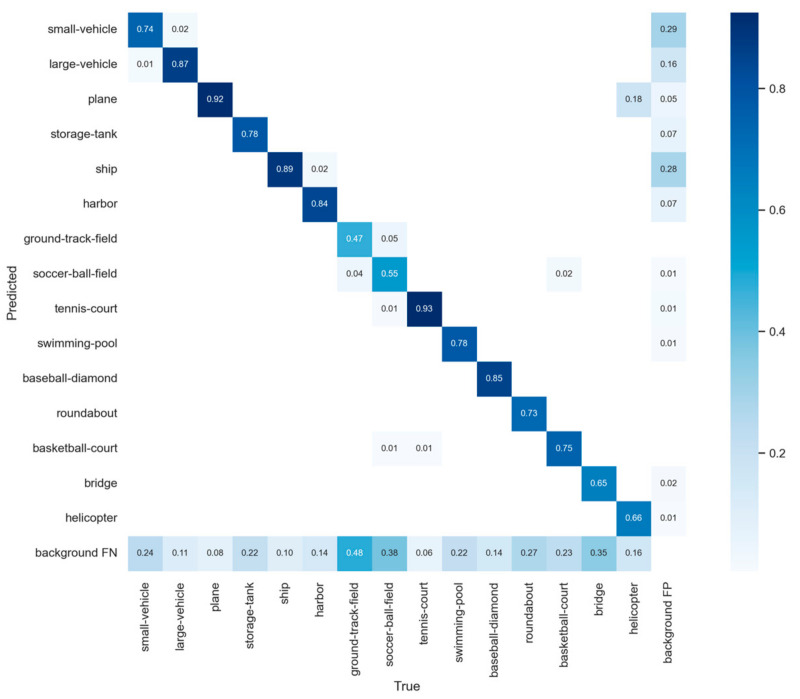
The confusion matrix from the proposed work on the DOTA dataset.

Then, we calculated the confusion matrix of proposed work in Figure 11. The confusion matrix can visualize the classification of each category. Each row represents the predicted categories, each column represents the actual categories, and the data on the diagonal line represents the proportion of categories that were correctly classified. However, the categories of ground track field (*GBF*), soccer ball field (*SBF*), and bridge (*BR*) had a high *FN*, which means that most objects of these classes will be missed in the process of detection. The corresponding *AP* was also very low. It is mainly because the number of objects in these categories were much less than other categories. The lack of training samples leads to a limited extraction of features and results in a high *FN*. In addition, the small vehicle and ship categories have a high *FP*, producing false alarms. Although the training samples of small vehicle and ship were sufficient, they belong to small objects, which are very hard to detect in a complex background.

To reflect the good properties of the proposed method, we compared the precision (P), recall (R), mean average precision (*mAP*), and inference time with those of YOLOv5s, YOLOv5m, YOLOv5l, and YOLOv5x. As shown in Table 2, compared to YOLOv5, the proposed work had a higher precision, recall and *mAP* due to the computational effect advantage of the Swin Transformer. However, the proposed work is slower than YOLOv5s because of the redundancy caused by the extra prediction head and Swin Transformer Block we added to improve the accuracy.

**Figure 12:**
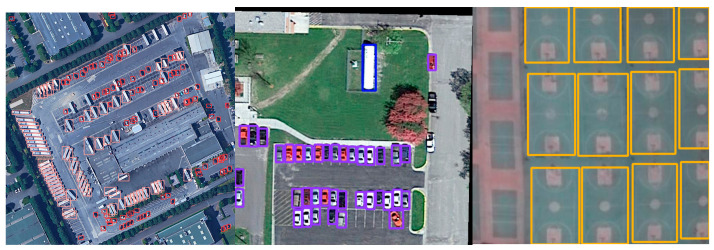
Some detection results of proposed work. Different colors show different classes.

To visually display the detection results of the algorithm, we selected some pictures to test from the DOTA dataset. As shown in Figure 12, the proposed work can identify most objects in the image, indicating that the improved algorithm has good recognition ability under complex background conditions in remote sensing images.

### 4.2. Ablation Experiment

We performed a series of ablation experiments on the DOTA dataset to explore the impact of each module on detection performance. We used the same training data and parameter settings to ensure the fairness and accuracy of the experiments. The results are presented in Table 3. “**√**” indicates that the method was used in the experiment, P2 indicates that four prediction heads were used for prediction, and SW-T indicates the CSPDarknet53 network with the Swin Transformer block.

Table 2 demonstrates that each module improved in this study had a certain improvement in the detection accuracy of the network. Improved K-means made the generated anchor boxes more suitable, and *mAP* increased by 1.4% without extra parameters. The C3SW-T module increased the number of parameters, but the *mAP* value and recall rate were significantly improved. To explore the influence of the *LN* (Layernorm) layer, we remove the *LN* layers in experiment 4. The result shows that the *mAP*, recall rate and precision were higher than with LN and reduced 0.02 M parameters. The extra prediction improved head parameters by 0.42 M, but the *mAP* increased by 0.7%. The Coordinate Attention mechanism increased the *mAP* by 1.1% with parameters increasing by only 0.06 M because the CA module fully utilizes long-range dependencies between spatial locations and captures location information better with almost no additional parameters. The weighted feature fusion to maximize the role of each feature map and improve its information representation capability of the feature map and the *mAP* improved by 2.4%. In conclusion, the proposed work increased the number of parameters from 7.2 M to 7.64 M, but detection performance was obviously improved, which increased *mAP* by 4.8 percentage points.

### 4.3. Comparison with Other YOLO Models

In this experiment, we compared the precision (*P*), recall (*R*), mean average precision (*mAP*), and inference time with some of the current mainstream YOLO series models at a 640 × 640 resolution. The experiment results are shown in Table 4. Among them, “proposed work-m” indicated the adoption of the same network depth as YOLOv5m.

As can be seen in Table 4, the proposed work was better than most of the current YOLO models. Because of the network depth, the proposed work was inferior to some lager models such as YOLOv5m6, but the proposed work did not differ significantly from them. When compared with the latest proposed works, YOLOv6 [30] and YOLOv7 [31], it was obvious that the proposed work achieved better results than YOLOv6s, YOLOv6l, and YOLOv7-tiny, but the inference speed was slower than them. Furthermore, when we adopted the same network depth as YOLOv5m, the detection performance has obviously improved to a level close to YOLOv7. This shows that there is still room for improvement in the model of YOLOv5s as the baseline model. Overall, proposed work outperformed most of the other YOLO models.

### 4.4. Scaled Image Size Exploration

To explore the impact of different resolutions on training results, we performed experimental training using YOLOv5m6, YOLOv6, YOLOv7, and the proposed work with a resolution of 1280 × 1280. The experimental results are shown in Table 4. As shown in Table 4, the proposed work achieved a higher accuracy than before and the gap between the latest YOLOV7 model was reduced. The reason is that remote sensing images are always lager than natural images and using larger scaled image sizes can retain more detailed feature information for small-object objection.

Through the comparison of Table 4 and Table 5, it can be found that selecting a model with a deeper network structure and a larger image resolution can effectively improve the detection accuracy. However, it should be noted that with the deepening of the network structure, the number of parameters to be calculated increases, and the inference speed decreases. Consequently, how to balance the accuracy and detection speed so that the detection model is most suitable for hardware deployment needs to be optimized in the improvement process.

### 4.5. Comparison with Other Models

To further quantitatively evaluate the performance of the proposed work, the HBB prediction task on the DOTA dataset was compared with other studies published in Table 3. Table 6 demonstrates that the algorithm in this study achieved the highest average precision value among all the compared algorithms and the best result among the fivecategories. Because of the network depth and the fact that some objects are too small and densely arranged, the accuracy of this study in the detection of some ground objects was slightly lower, but it did not differ significantly from other algorithms. Overall, the proposed algorithm outperformed the other methods, verifying its effectiveness.

## 5. Conclusions

For small-object detection in remote sensing images, current algorithms have low detection accuracy and inaccurate positioning. To address this issue, we designed a multi-scale feature fusion object detection algorithm based on the Swin Transformer and YOLOv5. First, *CIOU* was used as the similarity index to improve the K-means clustering algorithm to make the generated anchor boxes more suitable for the DOTA dataset. Second, we combined YOLOv5 with the modified Swin Transformer Block, and proposed an improved CSPDarknet53 structure to better capture global and local information. Then, we deepened the pyramid depth in the Neck part, and an extra prediction head was added for small-object detection. Regarding the path-aggregation Neck, a weighted bidirectional pyramid was used to improve the feature fusion network to better fuse the extracted shallow semantic features and deep semantic features and build a feature representation with rich location information and semantic information. Finally, Coordinate Attention was added to the YOLOv5 network to improve the detection accuracy of the multi-scale targets. The experimental results on the DOTA dataset showed that the *mAP* value, recall rate, and precision of the proposed method reached 74.7%, 80.4%, and 71.3, respectively, which significantly improved the detection accuracy and recall rate. In addition, comparative experiments were conducted using other advanced algorithms. The experimental results showed that the algorithm in this study achieved a high accuracy for small-target detection in remote sensing images. The proposed work is suitable for small-object detection in remote sensing images because of its high accuracy.

## Figures and Tables

**Table 1:** Accuracy comparison between YOLOv5 and proposed work.

Method	*PL*	BD	*BR*	GTF	SV	LV	SH	TC	BC	ST	*SBF*	RA	HA	SP	HC	*mAP*
YOLOv5s	89.9	73.2	44.2	47.2	62.2	82.3	87.0	93.1	64.1	69.4	46.0	56.8	75.6	60.2	**67.1**	67.9
Proposed work	**93.4**	**84.5**	**53.6**	**54.9**	**67.0**	**87.0**	**89.7**	**94.5**	**71.2**	**80.8**	**59.5**	**69.5**	**82.4**	**65.5**	66.6	**74.7**

**Table 2:** Accuracy comparison between YOLOv5 series and proposed work.

Method	Size	Precision (%)	Recall (%)	*mAP* (%)	Inference Time (ms)
YOLOv5s	640 × 640	75.95	60.27	67.9	**16**
YOLOv5m	640 × 640	79.29	61.67	68.5	34
YOLOv5l	640 × 640	78.80	63.13	69.7	49
YOLOv5x	640 × 640	77.86	64.45	70.1	57
Proposed work	640 × 640	**80.07**	**71.3**	**74.7**	32

**Table 3:** Test results after different improvement strategies are combined.

Improvement Strategy		Experiment						
		1	2	3	4	5	6	7
Improved K-means			**√**	**√**	**√**	**√**	**√**	**√**
C3SW-T (with LN)				**√**				
C3SW-T (without LN)					**√**	**√**	**√**	**√**
P2						**√**	**√**	**√**
Coordinate Attention							**√**	**√**
BiFPN								**√**
Evaluationindicator	*mAP* (%)	67.9	69.3 (+1.4)	70.6 (+1.3)	71.5 (+2.2)	72.1 (+0.6)	72.3 (+0.2)	**74.7 (+2.4)**
	Precision (%)	75.9	76.2 (+0.3)	77.8 (+1.6)	78.6 (+2.4)	79.1 (+0.5)	79.7 (+0.6)	**80 (+0.3)**
	Recall (%)	63.4	67.8 (+4.4)	68.3 (+0.5)	68.8 (+1)	69.2 (+0.4)	70.9 (+1.7)	**71.3 (+0.4)**
	Inference Time (ms)	16	16	23	21	27	29	32

**Table 4:** Accuracy comparison between YOLO series and proposed work.

Method	Image Size	Precision (%)	Recall (%)	*mAP* (%)	Inference Time (ms)
YOLOv5s6	640 × 640	78.7	66.9	71.0	29
YOLOv5m6	640 × 640	75.7	74.2	76.4	44
YOLOv6s	640 × 640	51.9	59.4	57.2	**10.5**
YOLOv6l	640 × 640	75.3	74.6	72.8	24.3
YOLOv7	640 × 640	**81.2**	**74.7**	**78.4**	18
YOLOv7-tiny	640 × 640	74.9	68	69.4	11
YOLOX-s	640 × 640	80.2	70	74.5	24
YOLOR-p6	640 × 640	79.7	61.2	64.7	20.7
Proposed work-m	640 × 640	79.4	74.1	77.3	47
Proposed work	640 × 640	80	71.3	74.7	32

**Table 5:** Accuracy comparison between YOLO series and proposed work.

Method	Image Size	Precision (%)	Recall (%)	*mAP* (%)	Inference Time (ms)
YOLOv5m6	1280 × 1280	79.4	77.3	77.2	58
YOLOv6s	1280 × 1280	58.5	62.6	63.1	34.1
YOLOv6l	1280 × 1280	75.3	**77.8**	71.9	33
YOLOv7	1280 × 1280	81.2	74.5	**80.4**	39.7
YOLOv7-tiny	1280 × 1280	74.6	68.5	70.1	**19**
Proposed work	1280 × 1280	**81.94**	72.4	78.1	51

**Table 6:** Comparison of the performance of other methods.

Method	*PL*	BD	*BR*	GTF	SV	LV	SH	TC	BC	ST	*SBF*	RA	HA	SP	HC	*mAP*
YOLOv4 [32]	93.8	77.1	42.3	42.9	**71.2**	70.9	88.3	94.5	55.8	68.7	42.6	39.4	77.3	**81.9**	74	68.4
YOLOv3 [33]	79.00	77.1	33.90	68.10	52.80	52.20	49.80	89.90	74.80	59.20	55.50	49.00	61.50	55.90	41.70	60.00
TPH-YOLOv5 [1]	91.8	77.7	49.5	68.1	66.6	84.7	87.2	93.7	64.7	69.7	53.1	62.7	**84.1**	63.3	53.9	71.4
ViT-YOLO [8]	**94.7**	79.2	48.8	60.7	68.4	72.7	89.1	**94.8**	58.8	70.2	53.1	57.9	84.0	77.8	**85.8**	73.1
SSD [4]	79.42	77.13	17.7	64.05	35.3	38.02	37.16	89.41	69.64	59.28	50.3	52.91	47.89	47.4	46.3	54.13
MDCF^2^Det [34]	89.68	84.39	52.12	72.71	64.49	67.07	77.45	90.11	**83.98**	**86.01**	55.03	63.33	74.45	67.74	62.87	72.86
FMSSD [35]	89.11	81.51	48.22	67.94	69.23	73.56	76.87	90.71	82.67	73.33	52.65	67.52	72.37	80.57	60.15	72.43
RoI-Transformer [36]	88.64	78.52	43.44	**75.92**	68.81	73.68	83.59	90.74	77.27	81.46	58.39	53.54	62.83	58.93	47.67	69.56
ICN [37]	90.00	77.70	53.40	73.30	73.50	65.00	78.20	90.80	79.10	84.80	57.20	62.11	73.45	70.22	58.08	72.45
DYOLO [38]	86.60	71.40	**54.60**	52.50	79.20	80.60	87.80	82.20	54.10	75.00	51.00	69.20	66.40	59.20	51.30	68.10
RetinaNet [39]	78.22	53.41	26.38	42.27	63.64	52.63	73.19	87.17	44.64	57.99	18.03	51.00	43.39	56.56	74.4	50.39
Proposed work	93.4	**84.5**	53.6	54.9	67.0	**87.0**	**89.7**	94.5	71.2	80.8	**59.5**	**69.5**	82.4	65.5	66.6	**74.7**

## Data Availability

Not applicable.
